# 4-Methyl­morpholinium bromide

**DOI:** 10.1107/S1600536810017447

**Published:** 2010-05-15

**Authors:** Ji-Yuan Yao

**Affiliations:** a Ordered Matter Science Research Center, College of Chemistry and Chemical Engineering, Southeast University, Nanjing 210096, People’s Republic of China

## Abstract

The six-membered ring in the title salt, C_5_H_12_NO^+^·Br^−^, has a chair conformation. In the crystal, the cations are linked to the anions by N—H⋯Br hydrogen bonds.

## Related literature

For background to phase transition materials, see: Hang *et al.* (2009 ▶); Zhang *et al.* (2009 ▶).
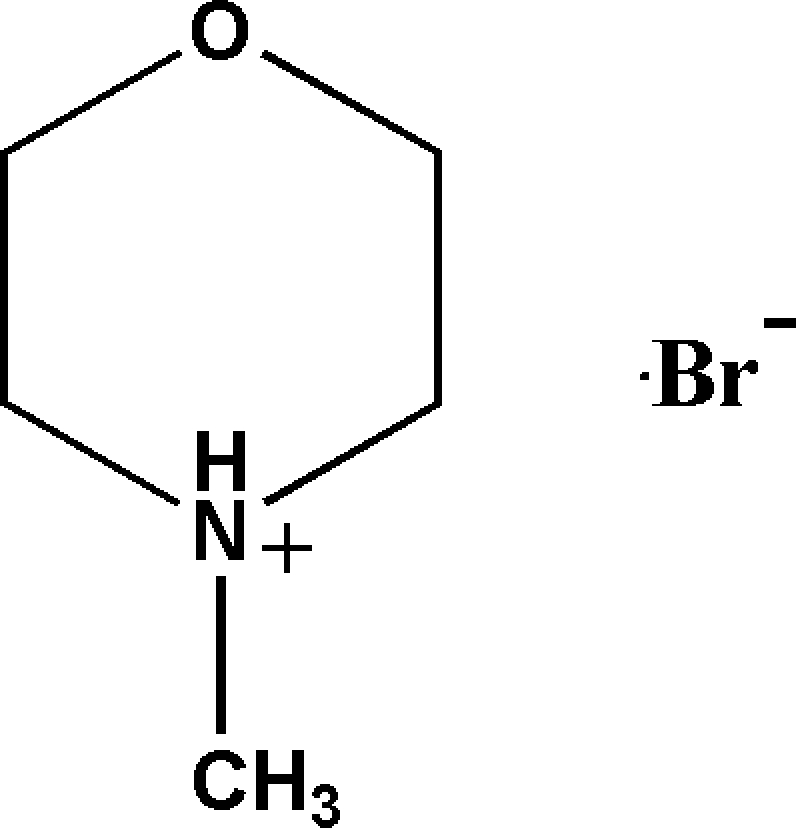


## Experimental

### 

#### Crystal data

C_5_H_12_NO^+^·Br^−^
*M* *_r_* = 182.07
Monoclinic, 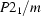

*a* = 7.3282 (15) Å
*b* = 7.4170 (15) Å
*c* = 7.3928 (15) Å
β = 92.72 (3)°
*V* = 401.37 (14) Å^3^
*Z* = 2
Mo *K*α radiation
μ = 5.04 mm^−1^
*T* = 293 K
0.40 × 0.30 × 0.20 mm

#### Data collection

Rigaku Mercury2 diffractometer
Absorption correction: multi-scan (*CrystalClear*; Rigaku, 2005 ▶) *T*_min_ = 0.178, *T*_max_ = 0.365
4192 measured reflections
995 independent reflections
866 reflections with *I* > 2σ(*I*)
*R*_int_ = 0.046

#### Refinement

*R*[*F* ^2^ > 2σ(*F* ^2^)] = 0.037
*wR*(*F* ^2^) = 0.098
*S* = 0.97
995 reflections
50 parameters
H-atom parameters constrained
Δρ_max_ = 0.33 e Å^−3^
Δρ_min_ = −0.72 e Å^−3^

### 

Data collection: *CrystalClear* (Rigaku, 2005 ▶); cell refinement: *CrystalClear*; data reduction: *CrystalClear*; program(s) used to solve structure: *SHELXS97* (Sheldrick, 2008 ▶); program(s) used to refine structure: *SHELXL97* (Sheldrick, 2008 ▶); molecular graphics: *SHELXTL*/*PC* (Sheldrick, 2008 ▶); software used to prepare material for publication: *PRPKAPPA* (Ferguson, 1999 ▶).

## Supplementary Material

Crystal structure: contains datablocks I, global. DOI: 10.1107/S1600536810017447/ng2770sup1.cif

Structure factors: contains datablocks I. DOI: 10.1107/S1600536810017447/ng2770Isup2.hkl

Additional supplementary materials:  crystallographic information; 3D view; checkCIF report

## Figures and Tables

**Table 1:** Hydrogen-bond geometry (Å, °)

*D*—H⋯*A*	*D*—H	H⋯*A*	*D*⋯*A*	*D*—H⋯*A*
N1—H1*B*⋯Br1	0.90	2.30	3.202 (4)	179

**Fig. 1.**
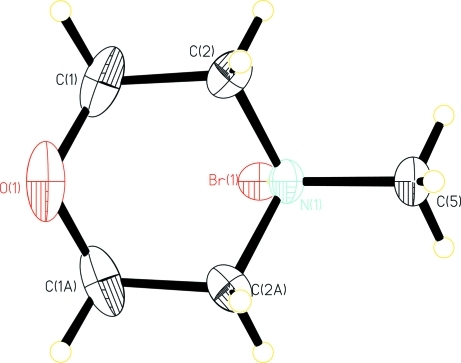
The molecular structure of the title compound, showing the atomic numbering scheme. Displacement ellipsoids are drawn at the 30% probability level, and all H atoms have been omitted for clarity.

**Fig. 2.**
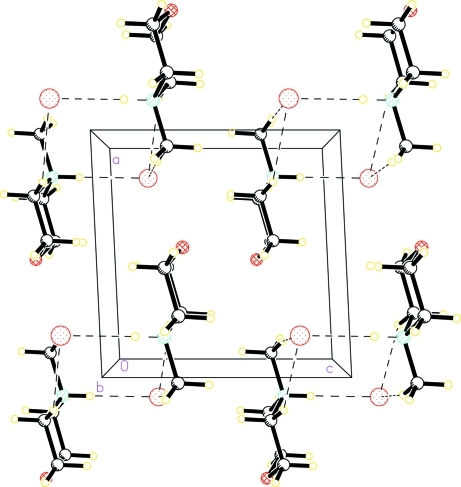
A view of the packing of the title compound, stacking along the b axis. Dashed lines indicate hydrogen bonds.

## supplementary crystallographic information

### Comment

As a study of phase transition materials, including organic ligands ( Hang *et al.* 2009 ), metal-organic coordination compounds (Zhang *et al.*, 2009 ), organic-inorganic hybrids, we studied the dielectric properties of the title compound, but there was no distinct anomaly observed from 90 K to 420 K, (m.p. 450 K) unfortunately. In this article, the crystal structure of (I) is showed.

The structure is composed of the *N*-Methylmorpholinium cations, hydrobromide anions (Fig. 1). in space group P21/m.

Packing structure of the title compound along b-axis are shown in Figure 2. *N*-Methylmorpholinium molecules are linked *via* hydrogen bonds of the type N—H···Br hydrogen bonds forming a two-dimensional planar sheets with hydrobromide anions. The hydrogen bonds are given in Table 1. The H atom of the protonated ring N atom (H1b) is donated to the Brl^-^ anions, being involved in a strong N—H···Br hydrogen bond. Br^-^ anions take part in electrostatics equilibrium with the *N*-Methylmorpholinium cations. The associated distances and angles are: Br···H—N 3.202 (4) Å, and 179.3°.

### Experimental

The title compound was prepared by reaction of stoichiometric amounts of *N*-Methylmorpholinium and concentrated hydrobromic acid in methanol. The obtained solution was filtered, and left at room temperature for 5 days. colorless crystals were obtained by slow evaporation.

### Refinement

Positional parameters of all the H atoms were calculated geometrically and were allowed to ride on the C, N atoms to which they are bonded, with *U*_iso_(H) = 1.2U_eq_(C),

*U*_iso_(H) = 1.2U_eq_(N).

### Crystal data

**Table:**

C_5_H_12_NO^+^·Br^−^	*F*(000) = 184
*M**_r_* = 182.07	*D*_x_ = 1.506 Mg m^−^^3^
Monoclinic, *P*2_1_/*m*	Mo *K*α radiation, λ = 0.71073 Å
Hall symbol: -P 2yb	Cell parameters from 0 reflections
*a* = 7.3282 (15) Å	θ = 3.8–27.5°
*b* = 7.4170 (15) Å	µ = 5.04 mm^−^^1^
*c* = 7.3928 (15) Å	*T* = 293 K
β = 92.72 (3)°	Prism, colourless
*V* = 401.37 (14) Å^3^	0.40 × 0.30 × 0.20 mm
*Z* = 2	

### Data collection

**Table:**

Rigaku Mercury2 diffractometer	995 independent reflections
Radiation source: fine-focus sealed tube	866 reflections with *I* > 2σ(*I*)
graphite	*R*_int_ = 0.046
Detector resolution: 13.6612 pixels mm^-1^	θ_max_ = 27.5°, θ_min_ = 3.8°
CCD_Profile_fitting scans	*h* = −9→9
Absorption correction: multi-scan (*CrystalClear*; Rigaku, 2005)	*k* = −9→9
*T*_min_ = 0.178, *T*_max_ = 0.365	*l* = −9→9
4192 measured reflections	

### Refinement

**Table:**

Refinement on *F*^2^	Primary atom site location: structure-invariant direct methods
Least-squares matrix: full	Secondary atom site location: difference Fourier map
*R*[*F*^2^ > 2σ(*F*^2^)] = 0.037	Hydrogen site location: inferred from neighbouring sites
*wR*(*F*^2^) = 0.098	H-atom parameters constrained
*S* = 0.97	*w* = 1/[σ^2^(*F*_o_^2^) + (0.0636*P*)^2^ + 0.1157*P*] where *P* = (*F*_o_^2^ + 2*F*_c_^2^)/3
995 reflections	(Δ/σ)_max_ < 0.001
50 parameters	Δρ_max_ = 0.33 e Å^−^^3^
0 restraints	Δρ_min_ = −0.72 e Å^−^^3^

### Special details

**Table:**

Geometry. All esds (except the esd in the dihedral angle between two l.s. planes) are estimated using the full covariance matrix. The cell esds are taken into account individually in the estimation of esds in distances, angles and torsion angles; correlations between esds in cell parameters are only used when they are defined by crystal symmetry. An approximate (isotropic) treatment of cell esds is used for estimating esds involving l.s. planes.
Refinement. Refinement of *F*^2^ against ALL reflections. The weighted *R*-factor wR and goodness of fit *S* are based on *F*^2^, conventional *R*-factors *R* are based on *F*, with *F* set to zero for negative *F*^2^. The threshold expression of *F*^2^ > σ(*F*^2^) is used only for calculating *R*-factors(gt) etc. and is not relevant to the choice of reflections for refinement. *R*-factors based on *F*^2^ are statistically about twice as large as those based on *F*, and *R*- factors based on ALL data will be even larger.

### Fractional atomic coordinates and isotropic or equivalent isotropic displacement parameters (Å^2^)

**Table:**

	*x*	*y*	*z*	*U*_iso_*/*U*_eq_	
O1	0.5268 (5)	0.7500	0.3399 (6)	0.0810 (14)	
N1	0.1476 (4)	0.7500	0.2465 (5)	0.0352 (7)	
H1B	0.1507	0.7500	0.1250	0.062 (16)*	
C1	0.4418 (5)	0.5939 (7)	0.2671 (6)	0.0749 (13)	
H1A	0.4478	0.5968	0.1377	0.096 (16)*	
H1C	0.5054	0.4884	0.3106	0.110 (18)*	
C2	0.2434 (4)	0.5854 (5)	0.3149 (5)	0.0488 (8)	
H2A	0.1864	0.4810	0.2604	0.054 (10)*	
H2B	0.2359	0.5765	0.4438	0.059 (11)*	
C5	−0.0464 (6)	0.7500	0.2948 (7)	0.0484 (11)	
H5A	−0.1058	0.6449	0.2455	0.061 (11)*	
H5B	−0.0534	0.7500	0.4234	0.059 (16)*	
Br1	0.15472 (6)	0.7500	−0.18624 (5)	0.0464 (2)	

### Atomic displacement parameters (Å^2^)

**Table:**

	*U*^11^	*U*^22^	*U*^33^	*U*^12^	*U*^13^	*U*^23^
O1	0.0332 (17)	0.143 (4)	0.066 (3)	0.000	−0.0037 (18)	0.000
N1	0.0288 (16)	0.047 (2)	0.0300 (17)	0.000	0.0003 (13)	0.000
C1	0.047 (2)	0.116 (4)	0.062 (3)	0.034 (2)	0.0023 (18)	−0.007 (3)
C2	0.0464 (17)	0.0512 (19)	0.0482 (19)	0.0130 (15)	−0.0023 (13)	−0.0017 (15)
C5	0.032 (2)	0.056 (3)	0.057 (3)	0.000	0.004 (2)	0.000
Br1	0.0608 (3)	0.0470 (3)	0.0314 (3)	0.000	0.0005 (2)	0.000

### Geometric parameters (Å, °)

**Table:**

O1—C1	1.409 (5)	C1—H1A	0.9600
O1—C1^i^	1.409 (5)	C1—H1C	0.9578
N1—C5	1.482 (5)	C2—H2A	0.9597
N1—C2^i^	1.485 (4)	C2—H2B	0.9596
N1—C2	1.485 (4)	C5—H5A	0.9562
N1—H1B	0.8997	C5—H5B	0.9550
C1—C2	1.514 (5)		
			
C1—O1—C1^i^	110.5 (4)	C2—C1—H1C	110.2
C5—N1—C2^i^	111.2 (2)	H1A—C1—H1C	108.0
C5—N1—C2	111.2 (2)	N1—C2—C1	109.3 (3)
C2^i^—N1—C2	110.6 (3)	N1—C2—H2A	109.3
C5—N1—H1B	108.1	C1—C2—H2A	109.9
C2^i^—N1—H1B	107.8	N1—C2—H2B	110.3
C2—N1—H1B	107.8	C1—C2—H2B	109.6
O1—C1—C2	110.9 (3)	H2A—C2—H2B	108.4
O1—C1—H1A	108.8	N1—C5—H5A	109.4
C2—C1—H1A	108.9	N1—C5—H5B	109.7
O1—C1—H1C	110.1	H5A—C5—H5B	109.5
			
C1^i^—O1—C1—C2	−61.8 (5)	C2^i^—N1—C2—C1	−54.4 (4)
C5—N1—C2—C1	−178.4 (3)	O1—C1—C2—N1	57.8 (4)

Symmetry codes: (i) *x*, −*y*+3/2, *z*.

### Hydrogen-bond geometry (Å, °)

**Table:**

*D*—H···*A*	*D*—H	H···*A*	*D*···*A*	*D*—H···*A*
N1—H1B···Br1	0.90	2.30	3.202 (4)	179

## References

[bb1] Ferguson, G. (1999). *PRPKAPPA* University of Guelph, Canada.

[bb2] Hang, T., Fu, D. W., Ye, Q. & Xiong, R. G. (2009). *Cryst. Growth Des.* **5**, 2026–2029.

[bb3] Rigaku (2005). *CrystalClear* Rigaku Corporation, Tokyo, Japan.

[bb4] Sheldrick, G. M. (2008). *Acta Cryst.* A**64**, 112–122. 10.1107/S0108767307043930 18156677

[bb5] Zhang, W., Chen, L. Z., Xiong, R. G., Nakamura, T. & Huang, S. D. (2009). *J. Am. Chem. Soc.* **131**, 12544–12545. 10.1021/ja905399x 19685869

